# First-in-Human Intratumoral Temperature Monitoring During Standard 3 T MRI Demonstrates RF-Induced Tissue Heating Within Clinical Safety Limits

**DOI:** 10.3390/bioengineering13070756

**Published:** 2026-06-28

**Authors:** Chie-Hee Cho, Franz Bergholz, Lutz Lüdemann, Carlo Bergholz, Emma Winger, Pauline Brand, Christian Spiegel, Wolfram Weschenfelder, Nikolaus Gaßler, Anna Xylander, Ingrid Hilger, Britt Wildemann, Gunther O. Hofmann

**Affiliations:** 1 Institute for Diagnostic and Interventional Radiology, Jena University Hospital, Friedrich Schiller University Jena, 07747 Jena, Germany; franz.bergholz@uni-jena.de (F.B.); pauline.brand@uni-jena.de (P.B.); 2 Department of Medical Physics, Clinic for Radiationtherapy, University Hospital Essen, 45147 Essen, Germany; lutz.luedemann@uk-essen.de; 3 Experimental Radiology, Jena University Hospital, 07747 Jena, Germany; carlo.bergholz@uni-jena.de (C.B.); ingrid.hilger@med.uni-jena.de (I.H.); 4 Experimental Traumatology, Jena University Hospital, 07747 Jena, Germany; emma.winger@uni-jena.de (E.W.); britt.wildemann@med.uni-jena.de (B.W.); 5 Department of Trauma, Hand and Reconstructive Surgery and Orthopedics, Clinic for Trauma, Jena University Hospital, 07747 Jena, Germany; christian.spiegel@med.uni-jena.de (C.S.); wolfram.weschenfelder@med.uni-jena.de (W.W.); gunter.hofmann@med.uni-jena.de (G.O.H.); 6 Section of Pathology, Institute for Forensic Medicine, Jena University Hospital, 07747 Jena, Germany; nikolaus.gassler@med.uni-jena.de (N.G.); anna.xylander@med.uni-jena.de (A.X.)

**Keywords:** magnetic resonance imaging, radiofrequency radiation, radiofrequency induced heating, intratumoral temperature measurement, hyperthermia, specific absorption rate, soft tissue sarcoma, thermometry, therapeutics

## Abstract

Magnetic resonance imaging (MRI) uses radiofrequency (RF) energy to generate diagnostic images. RF–tissue interactions lead to energy absorption and tissue heating, quantified by the specific absorption rate (SAR). Although SAR limits are strictly regulated for patient safety, actual in vivo tissue temperature changes during clinical MRI examinations in humans have not been directly measured. A patient with a histologically confirmed soft tissue sarcoma of the thigh underwent a clinically indicated 3 T MRI examination 24 h prior to resection. During imaging with whole-body SAR of 2.27 W/kg, direct temperature measurements (invasive and on the skin) were obtained. Temperatures increased by 2.0 °C within the tumor and at the skin surface was 3.4 °C at the skin surface. No technical difficulties or adverse events were observed, and the patient tolerated the examination well. This first-in-human case demonstrates the feasibility and safety of direct intratumoral temperature measurement during standard 3T MRI. While MRI was performed within safety limits of SAR as a surrogate for true tissue temperature, non-invasive temperature monitoring during MRI needs improvement. Controlled RF-induced heating during MRI may open new therapeutic possibilities, including MR-guided hyperthermia for sarcomas and other solid tumors or modulation of blood–brain barrier through transient RF-induced temperature elevations facilitating drug delivery.

## 1. Introduction

For magnetic resonance imaging (MRI), radiofrequency (RF) energy is essential for signal excitation. Electromagnetic alternating fields with frequencies above 10 MHz can induce perceptible tissue heating due to resistive (ohmic) losses. Physical models exist that simulate the temperature distribution induced by RF exposure under MRI conditions [1]. The models account for a thermoregulatory response, but do not consider different or individualized response. To date, it is generally assumed that the temperature curves follow a plateau pattern when exposed to RF energy.

The gold standard for assessing thermal effects is the direct temperature measurement. However, measuring temperature increases during MRI examinations is challenging. Therefore, the concept of the “specific absorption rate” (SAR)—defined as the rate of energy absorption per kilogram of bodyweight—is used as a surrogate to estimate RF-induced heating [2].

To prevent thermal injury, the International Electrotechnical Commission (IEC) has established specific safety limits (Table 1) [3]. Standard MRI exams are typically performed in normal mode, (SAR < 2 W/kg) or the first mode (SAR between 2 and 4 W/kg). SAR levels exceeding 4 W/kg are permitted only for research purposes and require prior approval by an ethics committee.

Temperature elevations caused by RF energy during MRI have been well documented in animal studies. However, in these studies, all animals were fully mechanically ventilated and under general anesthesia. As a result, the findings are not directly applicable to human MRI examinations, where most patients are fully awake and unsedated. An exception is a study in which pigs were allowed to breathe spontaneously and were only sedated and received analgesia [4]. The pigs were capable of hyperventilating—a thermoregulatory behavior not previously described in this species. Until then, pigs were known to regulate their body temperature primarily though wallowing. Additionally, changes in heart rate were observed that correlated to the increase in body temperature. For the first time, three additional temperature curve patterns—parabolic, sinusoidal, and linear—were identified alongside the previously known plateau-shaped curve. Hotspot temperatures ranging from 41.4 °C to 48.0 °C after 30 min of RF exposure with SAR levels ranging between 2.5 W/kg and 3.7 W/kg were observed, while maintaining a maximal increase in core body temperature of less than 1 °C (rectal end temperatures between 38.1 °C and 39.7 °C). After an additional 30 min of RF exposure, hotspot temperatures reached values between 42.2 °C and 55.9 °C [4]. These values correspond to the therapeutic temperature range targeted during hyperthermia treatment.

The physically calculated models of temperature distribution in humans under MRI conditions have certain limitations, as they do not fully account for the variability of individual thermoregulatory responses. This is particularly relevant as multiple forms of thermoregulatory response have been observed—regulated not only by heart rate and respiratory rate, but in humans also by blood pressure and sweating. Temperature elevation leads to systemic capillary dilation and, consequently, to changes in tissue perfusion. Therefore, direct temperature measurement—the gold standard—is essential for accurately determining actual tissue heating. To verify RF-induced temperature increase in humans during MRI examinations, an intervention involving invasive intratumoral temperature measurements was performed.

## 2. Materials and Methods

A 68-year-old female patient was diagnosed in August 2024 with a soft tissue sarcoma of the right thigh, measuring 66 × 83 × 103 mm^3^. Histological analysis revealed a pleomorphic sarcoma, staged as cT2b cN0 cM0 G3 (Stage IIIA). Her medical history included hypertension, type 2 diabetes mellitus, and hypothyroidism. Following MRI and CT imaging, the case was discussed in a multidisciplinary tumor board, where a consensus treatment strategy was established. The patient subsequently underwent preoperative radiochemotherapy, consisting of a total radiation dose of 50 Gy (5 × 2 Gy/week), combined with systemic chemotherapy: ifosfamide 1500 mg/m^2^ BSA/day on days 1–5 and 29–33, doxorubicin 50 mg/m^2^ BSA/day, on days 3 and 31, mesna 400 mg/m^2^ BSA, and G-CSF 48 Mio IE subcutaneously on days 6–8 and 34–36. After neoadjuvant therapy, the tumor progressed to a size of 112 × 121 × 160 mm. The patient met all eligibility criteria for the inclusion in the clinical study. This study was conducted in accordance with the principles of the Declaration of Helsinki. Ethics approval was obtained from the Ethics Committee of Jena University Hospital (approval number: 2024-3463-BO).

**Figure 1:**
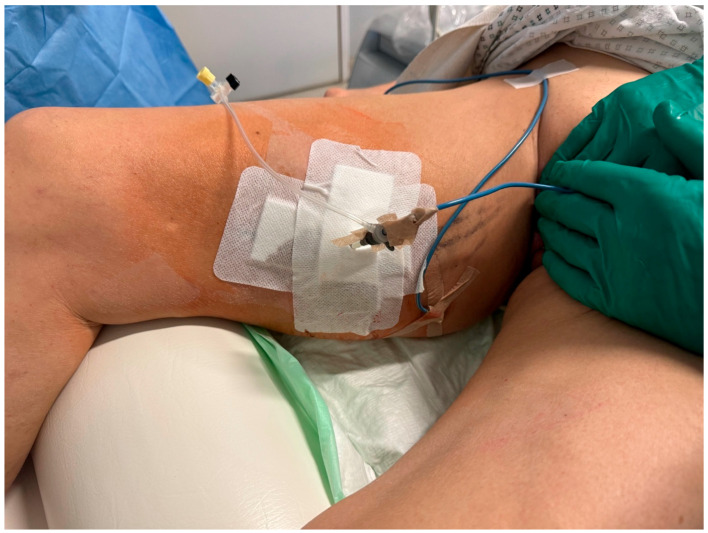
Position of intratumoral probe (blue) within the tumor, 8.5 cm from skin level, inserted through a 9 F sheath and secured with a white dressing. The position of the skin probe, fixed with brown dressing, is also shown.

After obtaining informed consent and prior to the MRI examination, the patient was prepared under sterile conditions and local anesthesia, and a fiber optic temperature probe (Opsens Solutions Inc., Quebec, QC, Canada; measurement range 20–45 °C) was inserted 8.5 cm from skin level under ultrasound guidance (Figure 1). The tip of invasive probe has a GaAs-crystal. The patient remained fully conscious and unsedated throughout the procedure. Vital signs, including blood pressure, heart rate, as well as oxygen and carbon dioxide levels, were continuously monitored (Tesla M3, Fa. MIPM, Frankfurt, Germany). Subsequently, the patient was positioned in a 3 T MRI scanner (Siemens Healthineers, Forchheim, Germany). The MRI scan consisted of 9 spin echo multiple-echos_15echos (duration 6 min) for heating and diffusion weighted imaging (5 min) for image evaluation over the course of 59.1 min.

**Figure 2:**
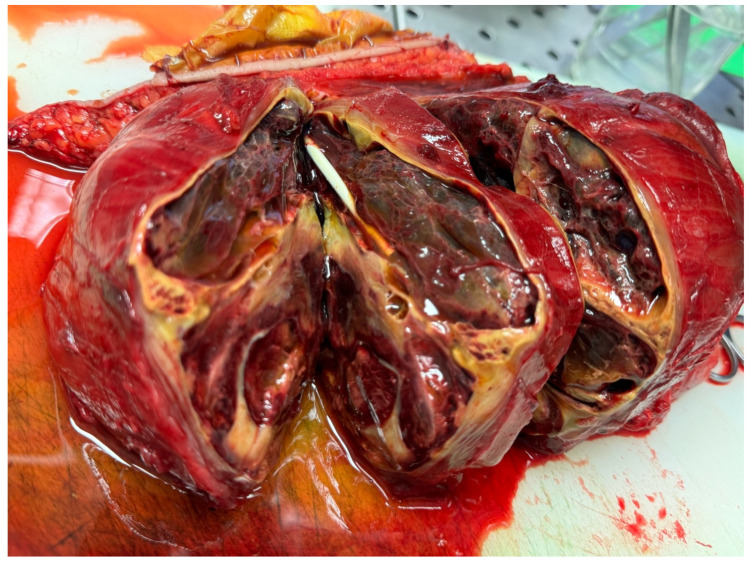
Position of the catheter sheath (white) within the dissected tumor.

Twenty-four hours after the intervention, the tumor was resected according to an R0-standard and the thermal reactions at the tissue level (Figure 2) could be evaluated.

## 3. Results

### 3.1. Temperature Results

#### 3.1.1. Temperature Changes Within the Body

**Figure 3:**
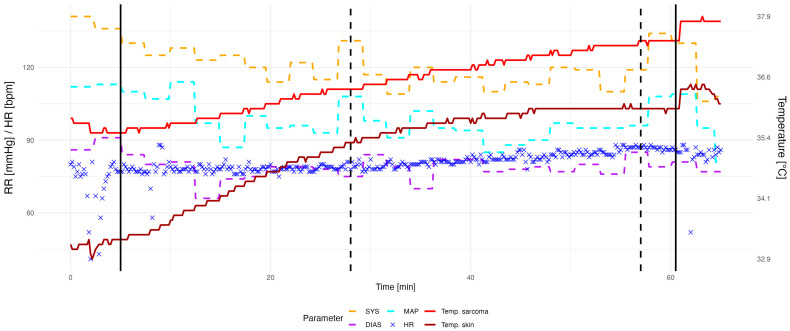
Vertical bold black lines indicate beginning and end of RF exposure. A minute before the first vertical bold black line a decrease in skin and sarcoma temperature was observed. After the second bold black line, indicating end of RF exposure, an increase of 0.42 °C is seen, as expected when the patient is moved out of the bore. Here the B0 decreases from 2.89 T to 1.6 T. The vertical dotted black lines mark the time points at which the patient reported a sensation of warmth, coinciding with an increase in systolic blood pressure (orange) and interpreted as a physiological response to thermal stress. After RF is turned off, the intratumoral temperature (red) shows no temperature change, while the temperature measured on the skin outside the tumor (brown, dashed line) decreases after RF was turned off, the increment when RF was turned off was due to transport of the patient out of the magnetic field. Mean blood pressure is shown in turquoise), and diasystolic blood pressure in purple. The heart rate is represented by the dotted blue curve.

**Figure 4:**
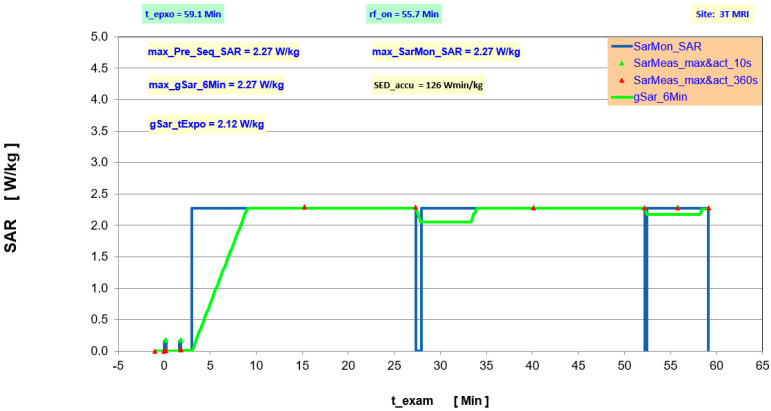
Whole-body specific absorption rate (SARwb) exposure during the MRI exam. The maximum SARwb recorded 2.27 W/kg. The specific energy dose (SED) was 126 Wmin/kg. SarMon_SAR and gSAR-6 Min are based on prediction while SarMeas_max&act {10 s, 360 s} are based on online measured values. Note: the red triangles partially overlap the green triangles.

Over a period of 59.1 min and whole-body SAR (SARwb) of 2.27 W/kg, the intratumoral temperature increased by 1.9 °C during the MRI scan and by 2.0 °C when measured outside the MRI (Figure 3). The specific energy dose (SED) was 126 Wmin/kg (Figure 4). The temperature increase on the skin was 3.1 °C during the MRI scan and 3.4 °C outside the MRI (Figure 3). All temperature curves followed a plateau trajectory.

#### 3.1.2. Temperature Changes Through the MRI Field

Another aspect is the change in temperature observed when the patient was moved into (first vertical bold black line) and out of the MRI bore (second vertical bold black line) (Figure 3). Outside the MRI bore the B0 field has about 1.6 T while inside the MRI bore it is 2.89 T. The intratumoral temperature decreased by 0.3 °C when entering the MRI bore and increased by 0.4 °C upon exiting (Figure 3). In contrast, the skin temperature decreased by 0.2 °C when entering and increased by 0.5 °C when exiting the MRI bore (Figure 3).

### 3.2. Physiological Results

As long as the systolic, diastolic, and mean arterial pressures moved in the same direction, the patient remained well during the MRI examination (Figure 3). During the episodes marked by the dotted lines at 24 and 56 min of RF exposure, a drop in diastolic pressure was observed, coinciding with the patient’s complaints of discomfort and a sensation of warmth. During the first episode, the discomfort was alleviated by removing the head covering and blanket. During the second episode, the examination was extended by additional three minutes through verbal reassurance. The sensation of discomfort resolved immediately upon deactivation of the RF signal.

Over the course of 59 min RF exposure (Figure 4), the average heart rate increased from 76 to 86 beats per minute (Figure 3; Table 2 both values measured outside the MRI). The temperaure increased intratumoral by 2°C and on the skin by 3.4°C, changes in blood pressures were observed over time.

## 4. Discussion

This case report is unique and not directly comparable to the existing literature. For example, Barber et al. (1990) exposed sheep to a 1.5 T MRI at a SAR of 4 W/kg for 20–105 min [5]; however, the animals were intubated and unable to breathe spontaneously like our patient, and temperature measurements were not obtained from the tumor tissue. In the study by Shrivastava et al. (2011), pigs were exposed to RF energy at 3 W/kg for three hours in a 7 T MRI scanner [6]—an exposure protocol that neither reflects the duration nor the RF conditions of standard human MRI examinations. Similarly, the experiments on dogs conducted by the group of Shuman et al. (1988) are not directly comparable to our case study [7]. Although the dogs were allowed to breathe freely—similar to awake human MRI patients—they were exposed to a SAR of 7.9 W/kg at 1.5 T for a duration of 27.6 mins. A shared limitation among all animal studies is that none of the investigated species possess the capacity for thermoregulation via sweating as humans do.

The models commonly used to calculate tissue temperature under RF exposure do not accurately reflect the actual measured values. Shrivastava et al. demonstrated that simulations using the empirical Pennes bioheat transfer equation were less accurate than those based on a mechanistic bioheat transfer model [8]. While the mechanistic bioheat transfer model better simulated in vivo temperatures in non-tumorous tissue, the reported increases—1.76 °C in the brain and up to 5.16 °C in hotspot regions in pigs—do not closely match the measured elevations observed in the present case: 1.9 °C intratumorally and 3.1 °C on the skin.

As the MRI was performed under first-level controlled operating mode (2–4 W/kg), with an actual SAR of 2.27 W/kg (Figure 4), both the intratumoral temperature (red curve) and skin temperature (dashed brown curve) remained within the defined safety thresholds (Figure 3). The fact that the observed temperature elevations did not reach the therapeutic hyperthermia range may be attributed to the excellent thermoregulatory capacity of the human body, including sweating and cardiovascular adjustments such as changes in blood pressure (orange, turquoise, and purple curves) and a slight increase in heart rate (dotted blue curve). These responses promote redistribution of blood flow and, consequently, influence heat dissipation. The recorded physiological responses probably account for the plateau in temperature rise (Figure 3).

The time points at which the patient reported discomfort in form of a sensation of warmth (dotted lines at 27 and 58 min after RF activation), her diastolic blood pressure dropped (purple curve) while systolic blood pressure (orange curve) continued to increase, findings that can be interpreted as physiological responses to thermal stress. Heart rate and blood pressure are important physiological parameters to consider during MRI examinations, as some patients experience anxiety or panic-like symptoms. Alterations in cardiovascular parameters during a resting and immobilized state may be perceived as unexpected and potentially contribute to patient discomfort.

Heat dissipation continued even after RF was turned off, as demonstrated by the decrease in skin temperature (dashed brown curve) and the stability of the intratumoral temperature (red curve) (Figure 3). The redistribution of heat from the hotspot to the whole body—resulting in an increase in core body temperature, as observed in the pigs [4]—could not be assessed in this case, as no rectal temperature probe was used.

Another interesting aspect is the change in temperature observed when the patient was moved into and out of the MRI core. The static magnetic field (B0) outside the MRI bore was 1.6 T and inside the bore was 2.89 T. The intratumoral temperature decreased by 0.3 °C when entering the MRI bore and increased by 0.4 °C upon exiting. In contrast, the skin temperature decreased by 0.2 °C upon entry and increased by 0.5 °C upon exit. According to Technical Report 01/2007 [9], it is known that fiber optic temperature probes exhibit measurement shifts when exposed to magnetic fields ranging from 1 T to 14 (measured in full Tesla increments). The temperature deviation (ΔT, in Kelvin) due to magnetic field strength (B0, in Tesla) follows the equation:

ΔT = 0.0603 − 0.1779 × B0 − 0.0351 × B02

According to the formula above, the temperature measurement should show a decrease of about than 0.7° when the patient is moved into the magnetic field at a strength of 2.89 T. The Siemens system operates at 2.89 T, which is conventionally rounded to 3 T. The formula applies only to fiber optic temperature sensors that utilize a GaAs crystal. However, temperature readings are influenced not only by the static magnetic field strength (B0) as shown in studies measuring between 2 T and 7 T, but also by differences in calibration conditions, i.e., whether calibration was performed inside or outside the MRI and under what reference temperature [10]. These factors introduce measurement errors in absolute temperature values (influenced by B0) and relative errors resulting from interactions between B0 and temperature itself.

In the analysis of the temperature increases, the plateau pattern—one of the four distinct thermoregulatory patterns (parabolic, sinusoidal, linear, and plateau) as described previously [4] and also known from hyperthermia treatment—was identified. This observation highlights the need for further investigations into heat development and thermoregulation in humans under MRI conditions.

Following chemoradiotherapy, tumor tissue undergoes changes on the three distinct levels. At the cellular level, these include apoptosis and necrosis, reduced cellular proliferation, and infiltration of inflammatory cells [11]. At the tissue architectural level, cell density decreases, accompanied by an expansion of the extracellular space and progressive fibrosis, ultimately resulting in tissue shrinkage [12]. In addition, radiotherapy affects the tumor microvasculature, leading to alterations in tumor perfusion and vascular integrity [13].

Hyperthermia is a well-established adjuvant treatment modality for various tumors, typically utilizing RF energy in the range of 70 to 100 MHz. Clinical indications include breast cancer, head and neck tumors, abdominal malignancies, and soft tissue sarcomas. Hyperthermia has been shown to improve survival rates [14], primarily through its synergistic effects with chemotherapy and radiation therapy, as well as its impact on cellular mechanisms [15]. Nevertheless, regional hyperthermia techniques still require further standardization and quality control [16]. Currently, hyperthermia is primarily offered in university-based centers and is not widely available in all regions—whereas MRI systems are readily accessible in most cities.

The present study provides the first intratumoral temperature measurements during MRI and may serve as a foundation for the future development of hyperthermia therapies using 3 T MRI. Traditional hyperthermia uses RF frequencies between 70 and 100 MHz; by comparison, the Larmor frequency at 1.5 T is approximately 64 MHz, and at 3 T it is approximately 128 MHz—suggesting that standard clinical MRI systems my inherently possess the physical capability to induce therapeutic heating.

This approach has the potential to be extended beyond currently established indications to include new areas, such as primary brain tumors and brain metastases. Hyperthermia is known to alter tumor perfusion, and a decrease in perfusion can lead to increased intratumoral temperatures [17], resulting in a heat steal effect that may explain the observed temperature rise.

Refinement of SAR limits may be necessary, as allowing higher SAR thresholds could enable the successful induction of therapeutic hyperthermia. Further advancements are required to optimize non-invasive temperature monitoring [8,18]. Proton resonant frequency (PRF) measurements were not performed, as they were beyond the scope of the present study. In addition, their acquisition would have considerably extended the examination time and may have resulted in a progressive decline in the intensity of energy deposition during the study. And strategies for the controlled modulation of thermoregulation need to be developed [19].

Further research is needed to accurately measure true tissue temperatures during MRI, with the dual aim of improving patient safety and exploring the full therapeutic potential of RF energy generated by MRI systems. Future studies should focus on determining which temperatures can be achieved at the upper range of the first-level controlled mode, without exceeding the SAR threshold of the first-level mode (4 W/kg).

## Figures and Tables

**Table 1:** Tolerated temperature increases due to RF exposure levels IEC 2015 [2].

Mode [W/kg]	Max. Local Tissue Temperature [°C]	Max. Core Body Temperature [°C]	Max. Increase in Core Body Temperature [°C]
Normal mode [<2 W/kg]	39	39	0.5
1. Level [2–4 W/kg]	40	40	1
2. Level [>4 W/kg]	>40	>40	>1

**Table 2:** Temperature increase—intratumoral 2.5 °C; on the skin 3.5 °C; decrease in heart rate and change in blood pressure. * measurement outside the MR.

Time RF Exposure [min]	Temperature Intratumoral [°C]	Temperature Skin [°C]	Heart Rate [Beats per min]	Blood Pressure (Systolic) [mmHg]	Blood Pressure (Diastolic) [mmHg]	Blood Pressure (Mean) [mmHg]
−1 min *	35.8	33.1	76	141	86	112
1 min	35.5	32.9	52	138	88	112
59 min	37.4	36.0	86	132	99	108
59 + 1 min *	37.8	36.5	84	106	77	95

## Data Availability

The datasets during and analyzed during the current study are available from the corresponding author on reasonable request.

## Abbreviations

ΔT	temperature difference
3T	3 Tesla
B0	static magnetic field strength
BSA	body surface area
CT	Computed Tomography
G CSF	granulocyte colony-stimulating factor
DIAS	diastolic blood pressure
F	French
Gy	gray
HR	Heart rate
IEC	International Electrotechnical Commission
MAP	mean arterial pressure
MHz	megahertz
MIPM	Mammendorfer Institut für Physik und Medizin GmbH
MRI	magnetic resonance imaging
RF	radiofrequency
RR	blood pressure
SAR	specific absorption rate
SarMeas	measured specific absorption rate
SARwb	whole-body specific absorption rate
SED	specific energy dose
SYS	systolic blood pressure
T	Tesla
Temp	temperature
W/kg	watt per kilogram
